# Temporal mechanically-induced signaling events in bone and dorsal root ganglion neurons after *in vivo* bone loading

**DOI:** 10.1371/journal.pone.0192760

**Published:** 2018-02-27

**Authors:** Jason A. Bleedorn, Troy A. Hornberger, Craig A. Goodman, Zhengling Hao, Susannah J. Sample, Ermias Amene, Mark D. Markel, Mary Behan, Peter Muir

**Affiliations:** 1 Comparative Orthopaedic Research Laboratory, School of Veterinary Medicine, University of Wisconsin-Madison, Madison, Wisconsin, United States of America; 2 Department of Comparative Biosciences, School of Veterinary Medicine, University of Wisconsin-Madison, Madison, Wisconsin, United States of America; 3 College of Health and Biomedicine, Victoria University, Melbourne, Victoria, Australia; 4 Australian Institute of Musculoskeletal Science (AIMSS), Victoria University, St Albans, Victoria, Australia; 5 Department of Medical Sciences, School of Veterinary Medicine, University of Wisconsin-Madison, Madison, Wisconsin, United States of America; University of California Davis, UNITED STATES

## Abstract

Mechanical signals play an integral role in the regulation of bone mass and functional adaptation to bone loading. The osteocyte has long been considered the principle mechanosensory cell type in bone, although recent evidence suggests the sensory nervous system may play a role in mechanosensing. The specific signaling pathways responsible for functional adaptation of the skeleton through modeling and remodeling are not clearly defined. *In vitro* studies suggest involvement of intracellular signaling through mitogen-activated protein kinase (MAPK), phosphatidylinositol 3-kinase (PI3K)/protein kinase B (Akt), and mammalian target of rapamycin (mTOR). However, anabolic signaling responses to bone loading using a whole animal *in vivo* model have not been studied in detail. Therefore, we examined mechanically-induced signaling events at five time points from 0 to 24 hours after loading using the rat *in vivo* ulna end-loading model. Western blot analysis of bone for MAPK’s, PI3K/Akt, and mTOR signaling, and quantitative reverse transcription polymerase chain reaction (qRT-PCR) to estimate gene expression of calcitonin gene-related protein alpha (CGRP-α), brain-derived neurotrophic factor (BDNF), nerve growth factor (NGF), c-jun, and c-fos in dorsal root ganglion (DRG) of the brachial intumescence were performed. There was a significant increase in signaling through MAPK’s including extracellular signal-related kinase (ERK) and c-Jun N-terminal kinase (JNK) in loaded limbs at 15 minutes after mechanical loading. Ulna loading did not significantly influence expression of the genes of interest in DRG neurons. Bone signaling and DRG gene expression from the loaded and contralateral limbs was correlated (S_R_>0.40, *P*<0.05). However, bone signaling did not correlate with expression of the genes of interest in DRG neurons. These results suggest that signaling through the MAPK pathway may be involved in load-induced bone formation *in vivo*. Further characterization of the molecular events involved in regulation of bone adaptation is needed to understand the timing and impact of loading events, and the contribution of the neuronal signaling to functional adaptation of bone.

## Introduction

Bone has the remarkable ability to continuously change shape and mass in response to a wide variety of mechanical loads [1]. This process has been described as functional adaptation primarily via a mechanism known as mechanotransduction, where cells sense physical stimuli, convert them into biochemical signals, and ultimately trigger a cellular response [2–4]. Osteocytes play a central role in this process due to their abundant distribution throughout bone, connections with other bone cells, and responses to stimulation or targeted ablation [5–7] (for reviews on osteocyte mechanotransduction [2,8,9]). Strain amplification must form part of the regulatory mechanism since physiological *in vivo* bone strain (<0.5%) is lower than that described to stimulate bone cells cultured *in vitro* (1–10% strain) [2,10–15]. Despite this adaptive ability, failure and associated accumulation of fatigue damage from cyclic loading of bone is common [13,16–18]. The precise cellular machinery that senses and enacts an adaptive response remains incompletely understood, thus increasing our knowledge in this area is important for comprehensive understanding of adaptative failure of bone.

Signaling events in bone have been studied using a variety of *in vitro* cell culture and *in vivo* animal models. *In vitro* osteocyte and osteoblast cell culture are designed to mimic physiologic events occurring with bone such as fluid flow, stretch, or deformation. Mechanical stimulation and fluid flow lead to upregulation of growth factors and activation of signaling pathways important for load-induced bone formation [2]. In particular, the phosphatidylinositol 3-kinase (PI3K)/protein kinase B (Akt) pathway and the mitogen-activated protein kinase (MAPK’s) are important for regulation of osteoblastic cell proliferation and differentiation [19–24]. These kinases then upregulate c-fos, c-jun, and activator protein-1 (AP-1) transcription factor which, in-turn, binds to mechanosensitive genes [25,26]. *In vivo* models are relatively non-invasive and allow for studying the entire bone/organ [27]. Measurement of bone strain, histomorphometry of bone accrual or loss in response to loading, unloading, or a pharmacologic treatment is typically determined using an *in vivo* model [28,29]. Bone tissue may also be evaluated using immunohistochemical staining or molecular techniques, such as *in situ* hybridization or gene expression, to study specific signaling pathways [28]. Despite the collection of knowledge on bone adaptation, more work is needed to thoroughly understand the inter- and intra-cellular signaling pathways that are activated by *in vivo* bone loading.

Load-induced bone formation has historically been regarded as a local site- and strain-specific phenomenon [30–32]. However, it has been proposed that a cross-talk mechanism enabling communication between bones within the skeleton exists [33,34]. Similar to osteocytes, sensory and sympathetic nerve fibers form a dense network in the periosteum and underlying bone tissue and, as such, are well positioned to sense changes in mechanical loading. Sensory fibers in bone may have regulatory effects on bone modeling and remodeling through release of neuropeptides and other signaling molecules into the bone microenvironment [34–37]. Neurotransmitters including calcitonin gene-related peptide (CGRP), brain-derived neurotrophic factor (BDNF), substance P (SP), vasoactive intestinal peptide (VIP) and neuropeptide Y (NPY) are released from these fibers to act on a wide variety of receptors on bone cells [38]. In particular, recent evidence by Tomlinson et al. suggests that mechanical signals increase nerve growth factor (NGF) expression in osteocytes, activating neurotrophic kinase receptor 1 (TrkA) sensory nerves, resulting in bone formation [39]. The entire mechanism by which *in vivo* bone loading potentially modulates neuronal networks and their impact on bone cell signaling remains to be determined.

Therefore, the purpose of this study was to examine the anabolic signaling events using a rat ulna end-loading model after *in vivo* bone loading, and determine if cross-talk between bones and the nervous system can be identified. Specifically, we investigated PI3K/Akt, MAPK, and mTOR signaling pathways in bone and determined the levels of CGRP-α, BDNF, *c-jun* and *c-fos* gene expression in DRG neurons in response to load. Bone loading resulted in an early increase in signaling through ERK and JNK; however, no corresponding effect in DRG neurons was identified for the candidate genes.

## Materials and methods

### Animals

A homogenous group of 67 young, male Sprague-Dawley rats (body weight, 296–376 g; age, 79±6 days) was used for the study. The rats were purchased from Harlan, Inc., and individually housed at the University of Wisconsin-Madison, School of Veterinary Medicine Animal Care Facility until the experiments were conducted. Rats were provided with food and water *ad libitum*.

### Ethics statement

All procedures performed in the experiments were conducted with the approval of the Animal Care & Use Committee, School of Veterinary Medicine, University of Wisconsin-Madison (V1148).

### Experimental design

Initially, a time course study was undertaken using 16 rats to determine the optimal time for analysis of mechanically-induced signaling events after ulna loading. Eight time points of 0, 15, 30 minutes, 1, 2, 6, 12, and 24 hours after loading were selected based on previous mechanical signaling events in other tissues and existing *in vitro* literature (n = 2 rats/time point). At each time point, the right ulna of one rat underwent mechanical loading and the second rat was sham-loaded. Rats were randomly chosen from the colony for group designation. An additional three rats were treated with insulin (5 IU/kg i.p., Humulin R, Novo Nordisk, Princeton, NJ) 15 minutes before euthanasia to serve as positive controls for the phosphorylation kinases in the PI3K/Akt/mTOR and MAPK signaling pathways [40].

Based on data from the time course study, time points of 15 minutes, and 1, 6, 12 and 24 hours after ulna loading were selected as the most informative regarding analysis of mechanically-induced signaling events after ulna loading. An additional 48 rats were then used for these time points. Load and sham group sizes were *n = 3*, respectively, at 15 minutes and 1 hour, and *n = 6* at 6, 12, and 24 hours.

### *In vivo* ulnar loading

**Fig 1:**
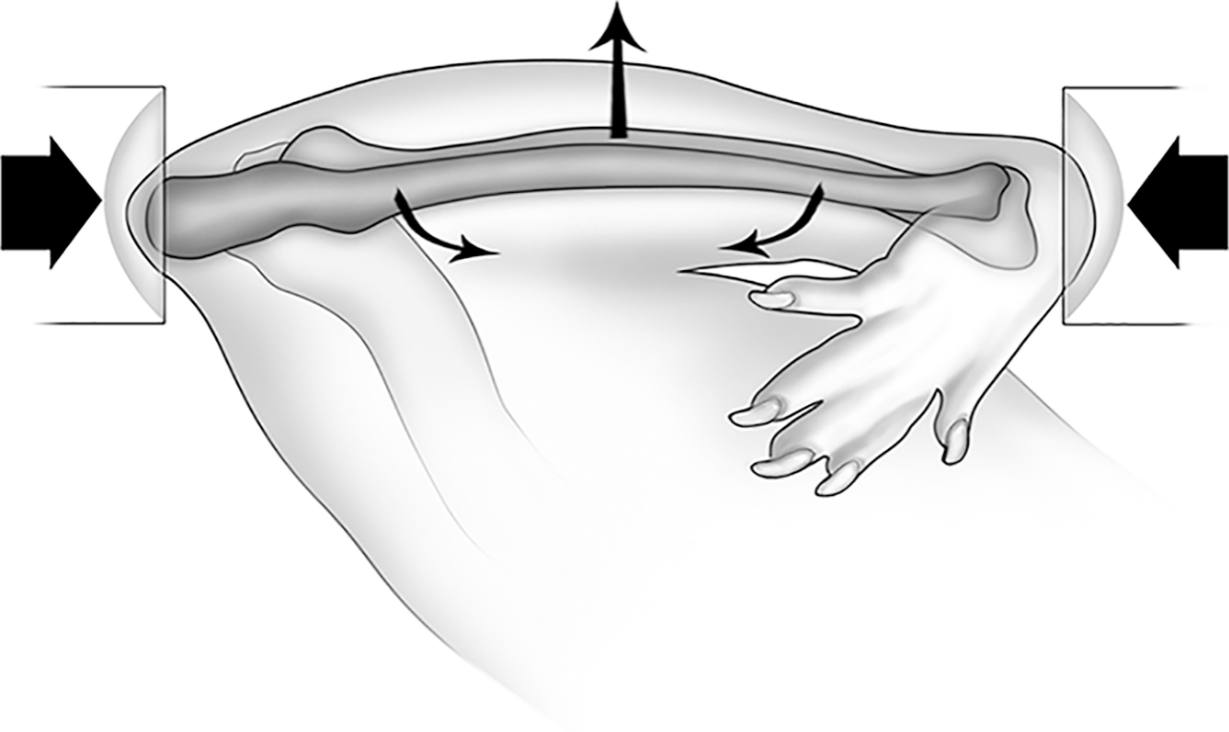
Schematic diagram of the rat ulna end-loading model. The right antebrachium was placed horizontally in loading cups attached to the materials testing machine. The medio-lateral diaphyseal curvature of the rat ulna is accentuated through axial compression, most of which is translated into a bending moment. Reproduced from [42] with permission from John Wiley & Sons.

*In vivo* loading of the right ulna was performed under isoflurane-induced anesthesia. Our group has previously used this model and shown that it induces functional adaptation in loaded and non-loaded bones [34]. Briefly, the right antebrachium of each rat was placed between horizontal oriented loading cups fixed to the loading platen and actuator of a materials testing machine (Model 8800 DynaMight, Instron, Canton MA, USA) using a 250 N load cell (Honeywell Sensotec, Canton, MA, USA) (Fig 1). The distance between the actuator cup and the load cell cup was manually adjusted until −0.5 N was applied to the ulna to hold it in position. Axial compression of the antebrachium accentuates the natural mediolateral curvature of the ulnar diaphysis, translating the compressive force into a bending moment maximal near the mid-shaft [41,42]. The relationship between peak load and initial peak strain in this rat end-loading model was previously determined [34]. Cyclic compressive load was applied to the right antebrachium for 1,500 cycles at a peak strain of -3750με using a haversine waveform at 4Hz and a peak load of -18.0N. The single period of *in vivo* loading lasted for 6.25 minutes. Glycopyrrolate (0.01 mg/kg sc) was given 15 minutes before induction. For analgesia, butorphanol (0.5 mg/kg sc) was given 15 min before induction and again immediately after loading. Rats were ambulatory within 20 min of recovery from anesthesia.

### Tissue sample collection

Tissue samples were collected under terminal general anesthesia. The right and left ulnae were excised, bone ends were cut to remove the articular surfaces, and cold PBS-soaked gauze was used to remove remaining adherent musculature from the bones leaving the periosteum intact. Collection time and handling of bones was minimized (<1 minute) to ensure consistency among groups. Bones were placed in 2ml microtubes, and immediately frozen in liquid nitrogen. Ulnae were stored at—80°C. Each frozen bone was ground under liquid nitrogen using a mortar and pestle, then homogenized with a Polytron in a buffer containing 40mM Tris (pH 7.5), 1mM EDTA, 5mM EGTA, 0.5% Triton X-100, 600mM β-Glycerolphosphate, 1M NaF, 1M Na_3_VO_4_, 10mg/ml Leupeptin, and 0.2M Phenylmethanesulfonyl Fluoride (PMSF). Homogenates were centrifuged at 4,000 g for 5 min and the supernatant was saved for analysis at -80°C.

After excision of the ulnae, rats were prepared for perfusion and collection of dorsal root ganglia (DRG) under terminal general anesthesia. The heart was approached by median sternotomy and a 22-gauge inflow needle was placed in the left ventricle. The right auricle was then incised for egress flow. Approximately 50ml of sterile 0.9% NaCL was perfused into the circulation until fluid flow from the egress needle was clear. Rats were then perfused with 75ml of RNA*later®* solution (Sigma-Aldrich, St. Louis, MO). A continuous dorsal laminectomy from C_1_-T_2_ was made to expose the dorsal nerve roots of the spinal cord. Right and left DRG were collected from the brachial intumescence (C_6_-T_2_). DRG were placed in tubes containing 2ml of RNA*later®*. The brain was exposed using a combined dorsal-suboccipital craniectomy. Right and left trigeminal ganglia were excised and placed in tubes containing RNA*later®*, to serve as an internal control tissue from a ganglion receiving sensory input that is not associated with a limb.

### Western blot analysis of bone tissue

Markers for signaling through mTOR [P-p70^s6k^(389) and total p70], PI3K [P-PKB(473) and P-PKB(308)], and MAPK’s [P-JNK(183/185) and P-ERK(202/204)] were evaluated at several time points in bone. Supernatant protein concentration of each sample was determined by the DC protein assay (Bio-Rad Laboratories, Hercules, CA), and equivalent amounts of protein from each sample were analyzed on a Western blot as previously described [43]. Briefly, samples were dissolved in Laemmli buffer and subjected to electrophoretic separation by SDS-PAGE on 7.5% acrylamide gels. After separation, proteins were transferred to a PVDF membrane, blocked with 5% powdered milk in Tris-buffered saline, 1% Tween 20 (TBST) for 1 hour, washed for 10 minutes in TBST, followed by an overnight incubation at 4°C with primary antibody. After overnight incubation, membranes were washed for 30min in TBST and then probed with an anti-rabbit antibody in 5% milk TBST for 45 min at room temperature. Primary antibodies for P-JNK(183/185) (#4671S), P-ERK(202/204) (#9101S), P-PKB(473) (#9271S), P-PKB(308) (#9275S), and total p70 (#2708) were obtained from Cell Signaling (Danvers, MA). The primary antibody for P-p70S6k(389) (#11759) was obtained from Santa Cruz Biotechnologies (Santa Cruz, CA). The peroxidase-conjugated anti-rabbit antibody (#PI-1000) was obtained from Vector Laboratories (Burlingame, CA). After 30 min of washing in TBST, the blots were developed on film using ECL (Pierce, Rockford, IL, USA) or ECL Plus (Amersham, Piscataway, NJ). Images were captured using conventional exposure radiography. Select membranes were rinsed, antibodies removed with stripping buffer (Restore, Thermo Fisher Scientific, Rockford, IL), and re-incubated with a different primary antibody and processed as described above. At the end of the Western blot analyses, the PVDF membranes were stained with Coomassie Blue to verify equal loading of protein in all lanes. Densitometric measurements of each blot were carried out with ImageJ (NIH, Bethesda, MA).

### qRT-PCR for DRG gene expression

RNA was isolated from DRG using TRIZOL (Invitrogen, Carlsbad, CA). Total RNA was purified using standard RNA clean-up reagents (Invitrogen, Carlsbad, CA). cDNA was prepared from total RNA by using the Superscript III first-strand synthesis system (Invitrogen, Carlsbad, CA). A melt-curve analysis was performed to check for primer-dimer formation. qRT-PCR was performed for expression of CGRP-α, BDNF, NGF, *c-jun* and *c-fos* using standard Taqman primers (Assay-on-Demand, Applied Biosystems); 18S rRNA was used as the housekeeping gene (Table 1). The additional TaqMan oligonucleotide probe was required to anneal between the two primers in order to generate a fluorescent signal. Duplicate PCR reactions were performed and internal cDNA controls were used to check that C_t_ values are consistent between assays. Gene expression in right and left trigeminal ganglia were used as internal controls. Analysis of DRG qRT-PCR data was performed using the 2^−ΔΔCT^ method [44].

**Table 1:** Oligonucleotide primers for quantitative real-time reverse-transcriptase-polymerase chain reaction.

mRNA Targets	Primer Type	Oligonucleotides (5’ to 3’)	Amplicon Size (bp)	Sequence Reference
BDNF	Forward	AAAACCATAAGGACGCGGACTT	22	Rapanelli et al. 2010 [45]
	Reverse	AAAGAGCAGAGGAGGCTCCAA	21	
CGRP-α	Forward	GCATGGCCACTCTCAGTGAAG	21	Laboratory of Dr. Muir, University of Wisconsin-Madison
	Reverse	CCTGACTTTCATCTGCATATAGTTCTG	27	
NGF	Forward	TGCATAGCGTAATGTCCATGTTG	23	Squillacioti et al. 2009 [46]
	Reverse	CTGTGTCAAGGGAATGCTGAA	21	
c-fos	Forward	TCCACTGCCTGGGACAGAA	19	Rapanelli et al. 2010 [45]
	Reverse	CGCAGCGATCTTCATCAAAC	20	
c-jun	Forward	CGGCCCCGAAACTTCTG	17	Rapanelli et al. 2010 [45]
	Reverse	GTCGTTTCCATCTTTGCAGTCA	22	
18S rRNA	Forward	CGCCGCTAGAGGTGAAATTCT	21	Laboratory of Dr. Svaren, University of Wisconsin-Madison
	Reverse	CGAACCTCCGACTTTCGTTCT	21	

### Statistical analysis

Statistical analyses were performed using GraphPad Prism (Version 6.00, La Jolla, CA, USA). The Kolmogorov-Smirnov test was used to determine whether sample data were normally distributed. All values are expressed as mean ± standard deviation. The right ulna of the sham-loaded animal was used as control for evaluation of mechanical loading effects. The effect of loading was analyzed using the Student’s *t* test for unpaired data for comparison of the loaded limb (right) in the load group with the right limb in the sham group. In the load group, the loaded and contralateral limbs were also compared using the Student’s *t* test for paired data. The Spearman rank correlation statistic (S_R_) was used to determine whether changes in bone signaling after load was correlated with DRG gene expression. Data were assessed at individual time points and also combined to evaluate the effect of load or limb for correlation analysis. Differences between groups were considered significant when *P*<0.05.

## Results

We developed an *in vivo* whole animal model that enables determination of the effects of mechanical loading on various signaling events in bone and nerve tissue. Protein extraction from each ulna yielded a concentration of 3.74±1.6μg/μl, which was sufficient to perform western blot analysis for the signaling pathways of interest. Protein extraction from DRG neurons did not yield an adequate quantity of protein (0.5–1.3μg/μl); therefore, gene expression was estimated using qRT-PCR from DRG tissue.

### Effect of mechanical loading on protein kinase phosphorylation

**Fig 2:**
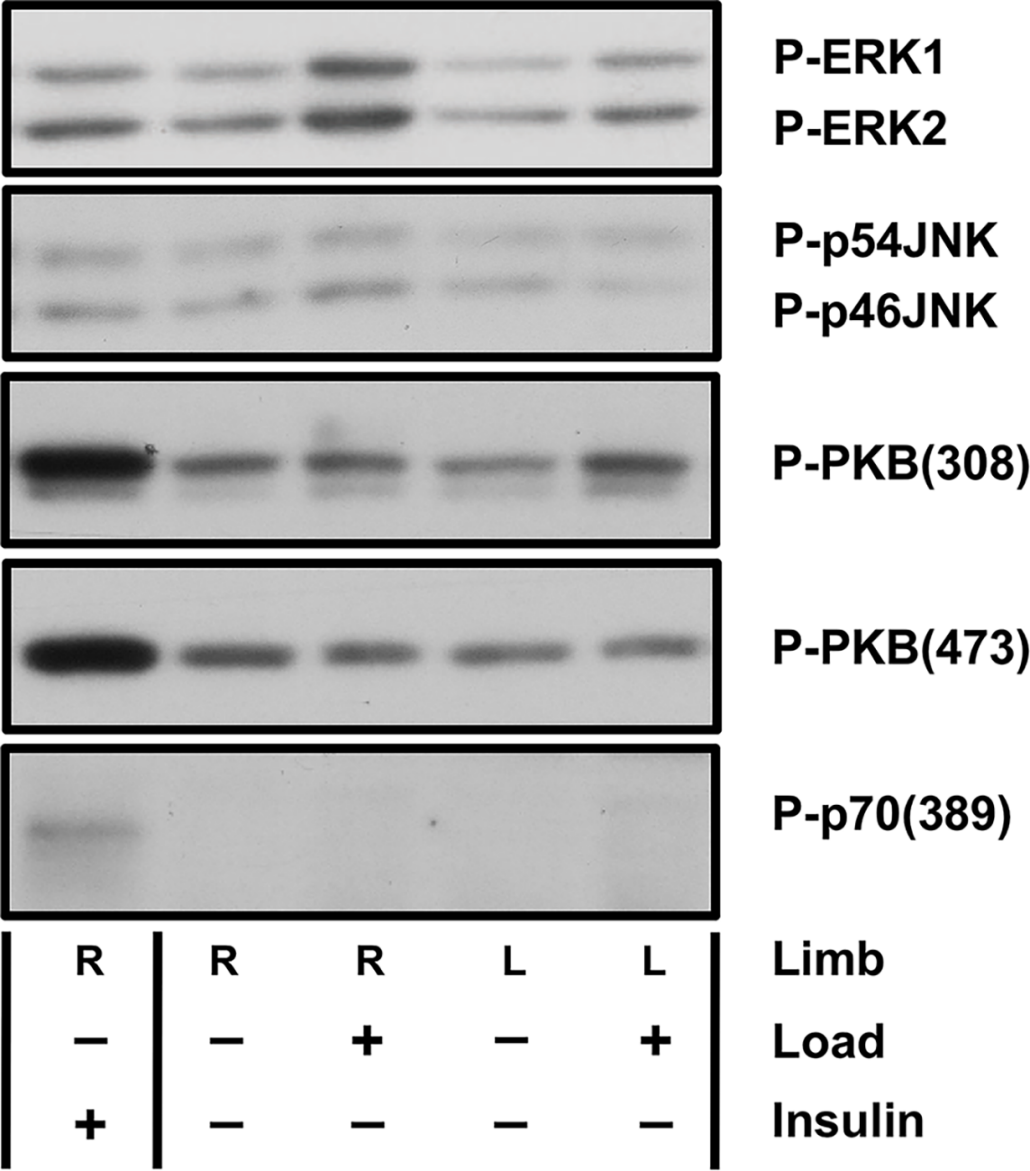
Representative Western blots for bone signaling at 15 minutes after loading. Insulin treatment served as positive control. Paired right and left limbs from Sham and Loaded rats were compared (n = 3/group). Phosphorylation of ERK and JNK were significantly increased in the loaded right ulnae at 15 minutes after loading compared to sham and contralateral limbs. The upper band was quantified for P-p54JNK.

**Fig 3:**
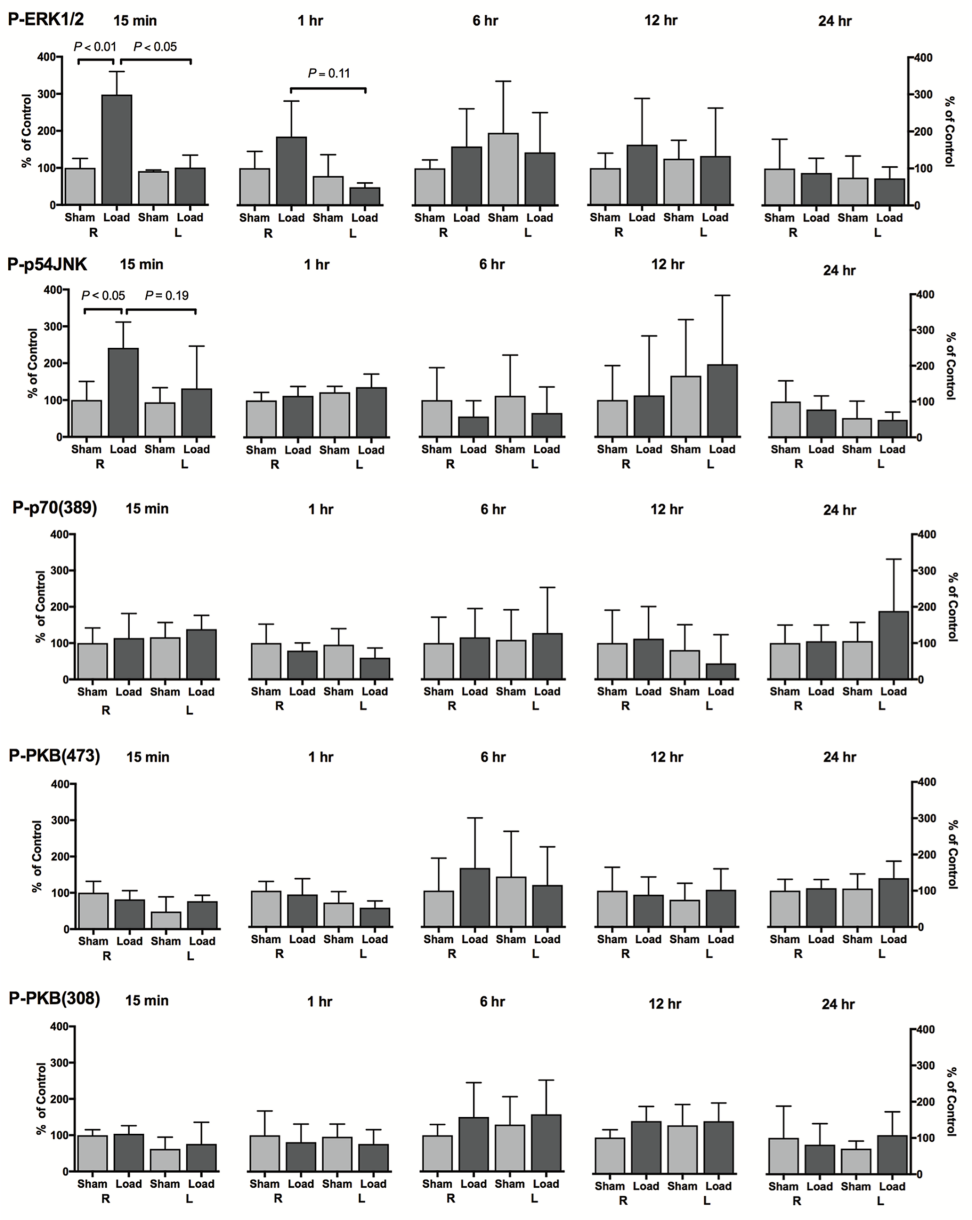
Signaling events in bone after a single period of mechanical loading. A cyclic compressive load was applied to the right antebrachium for 1,500 cycles at a peak strain of −3750με using a haversine waveform at 4Hz and a peak load of −18.0 N. Significant increases were noted in phosphorylation of ERK and p54JNK in the right loaded ulnae compared to sham controls as measured by Western blot analysis. All data is expressed as a percentage of the right sham control limb.

Mechanical loading resulted in a cascade of protein kinase phosphorylation events evident early after loading (Figs 2 and 3). Phosphorylation of ERK (P-ERK1/2) was significantly increased in the loaded ulnae at 15 minutes after loading (Fig 3A) compared to sham and contralateral limbs. A trend for activation of signaling through ERK was evident in loaded ulnae compared to contralateral limbs at 1 hour (*P* = 0.12) (Fig 3A). Activation of ERK signaling was not significantly different at any later time points. Phosphorylation of JNK (P-p54JNK) was significantly increased in the loaded ulnae at 15 minutes after loading compared to sham limbs (Fig 3B); however, no difference was noted at any later time points in either limb. No significant effects of load were identified in the PI3K/Akt/mTOR pathway [P-p70(389), P-PKB(473), or P-PKB(308)] in loaded or contralateral limbs (Fig 3C).

### Gene expression in DRG neurons

No significant differences in gene expression in response to loading were noted for CGRP-α, BDNF, *c-jun* or *c-fos* at any time point in DRG from loaded limbs. Trends were noted at 12 hours after loading in contralateral limbs for higher expression of BDNF (*P* = 0.09) and CGRP-α (*P* = 0.19).

### Correlation of bone signaling and DRG neuron gene expression

Bone signaling and DRG neuron gene expression from loaded right and contralateral left limbs were correlated (S_R_ = 0.40–0.73, *P*<0.05). There were mild positive correlations between PKB308 and PKB 473 phosphorylation in bone (Table 2). In loaded limbs, DRG neuron *c-*fos expression was correlated with both CGRP and BDNF gene expression. However, no significant correlations were found between bone signaling and expression of the panel of candidate genes of interest in DRG at the time points examined (Table 2).

**Table 2:** Correlation of bone signaling activity and DRG gene expression in loaded limbs. There were mild positive correlations between PKB308 and PKB473 bone signaling. In loaded limbs, expression of genes examined was related. No correlations were found between bone signaling and DRG gene expression. Spearman rank (S_R_) correlation values listed for combined loaded right and left limbs. Shading represents significant correlations, darker indicating stronger correlation value.

p70	JNK	ERK	PKB473	PKB308	BDNF	CGRP	*c*-fos	*c*-jun	NGF	
	-0.24	0.17	0.44	0.10	-0.18	-0.24	-0.51	-0.22	-0.24	p70
		0.32	-0.22	0.11	0.00	0.09	0.14	-0.12	0.16	JNK
			0.20	0.39	0.03	0.02	0.06	0.10	0.09	ERK
				0.46	0.02	-0.05	0.18	0.31	-0.16	PKB473
					0.19	0.15	0.10	0.39	-0.09	PKB308
						0.86	0.53	0.82	0.51	BDNF
							0.53	0.76	0.49	CGRP
								0.79	0.63	*c*-fos
									0.42	*c*-jun
										NGF

## Discussion

We demonstrate the ability to detect signaling events in bone tissue *in vivo* using the rat ulna end-loading model and Western blot analysis. We also quantified downstream products using qRT-PCR to estimate expression of a panel of candidate genes in spinal cord DRG thought to be involved in bone functional adaptation. Load-induced bone formation was associated with phosphorylation of several protein kinases, evident at different time points after mechanical loading in rats. Activation of MAPK signaling (P-ERK and P-JNK) appears rapidly after loading and diminishes rapidly over time. However, PI3K/Akt/mTOR pathway [P-PKB and P-p70(389)] signaling did not appear to be altered by the loading protocol employed in this study. Similarly, no significant changes were identified in our panel of candidate genes associated with neuronal signaling, or in correlations with bone kinase signaling.

Bone has a remarkable ability to alter its mass and architecture in response to changes in its local environment. Various mechanical stimuli have been shown to influence bone cell metabolism and activate signal transduction in bone cells *in vitro*, including the MAPK’s, WNT/β-catenin, calcium ions, integrins, nitric oxide and prostaglandins [47]. Limited data describing signaling events in living tissue exists. Most studies have used *in vitro* models studying manipulation of the mechanical environment of osteoblasts and osteocytes in cell culture, or immunostaining of bone tissue after mechanical stimulation [28,48]. Use of an *in vivo* model to investigate signaling responses in bone tissue in response to loading is likely to be important to future work. Loaded and non-loaded bones were analyzed at various times after a mechanical stimulus that has been shown to induce functional adaptation in multiple bones and was based on previous work in our laboratory [34]. Bone tissue was prepared to limit iatrogenic activation of signaling molecules. Signaling responses were examined in pathways important for bone adaptation by Western blot analysis, normalized to control tissue of similar time points after sham loading. This technique yielded adequate protein concentration from the ulnas of rats, and we could identify signaling events in all pathways examined. The time points chosen for the study were based on pilot data, as prior knowledge of the timeline of mechanically induced signaling events in bone *in vivo* is very limited. We expected that some signaling events would be very rapid. Investigation of additional time points, particularly later times, may be informative with regards to signaling responses in non-loaded bones. Alternative loading regimens, including different levels of peak strain, different numbers of load cycles, periods of rest [49], or repeated bouts of loading, have been shown to impact molecular responses and load-induced bone formation [49,50], and would have been interesting to examine using this model.

In the present study, the most consistent and notable impact of mechanical stimulation was increased signaling through JNK and ERK shortly after loading. ERK, JNK, and p38 are the best studied members of the MAPK family and have a central role in regulation of bone mass. MAPK’s mediate bone formation through differentiation of both osteoblasts [51] and osteocytes [8,21,52]. ERK, in particular, regulates a wide breadth of transcription factors including early (RUNX2) and late (ATF4) stage osteoblast differentiation, suggesting the role may vary based on stage and anatomic location [51]. ERK signaling is episodic in nature [53]. and returns to basal levels after prolonged loading [54], which may impact detection over a time course study.

The lack of a load effect on signaling through the PI3K/Akt/mTOR pathway was unexpected. *In vitro* work suggests that Akt signaling is one of several key pathways important for bone cell differentiation, function, and survival [55]. A trend for increased signaling activity was observed in p70(389) and P-PKB(473) at 12 and 24 hours after load during the initial time course portion of the experiment. However, this response was not consistent when additional animals were studied at these time points.

Identification of significant loading effects was influenced by data variation within individual time points and signaling pathways. Mechanical signaling machinery can be exquisitely sensitive to any iatrogenic stimulus. Although mechanical loading, tissue collection and processing was efficient and meticulous, variation in bone collection technique or time may have contributed to data variance as some variation in the concentration of protein extracted from the ulna of similar sized rats found. Load-induced bone formation in the rat ulna after end-loading is greatest in the mid-diaphysis where strain is concentrated [41,56]. Peak strain is experienced 1-2mm distal to the midpoint of the ulna and reduces substantially along remaining the ulnar length of the bone [57]. The entire ulna was used for this work, to maintain consistency, ease of tissue collection, and ensure an adequate tissue volume for analysis. It is possible that use of the entire ulna may have reduced the signaling response detected in our study. Protein isolation from only the mid-diaphysis of the ulna may aid the detection of significant loading effects. Alternative strategies such as *in situ* hybridization may provide support in correlation of a molecular response to a tissue level response.

Early signaling events were only detected in loaded bones. Similarly, the pattern of bone signaling and DRG neuron gene expression was similar between right and left limbs of individual animals for all pathways and genes studied. A neuronal mechanism is one possibility that may be responsible for such effects [33,34]. To begin to address this hypothesis, we quantified expression of candidate genes in DRG from the thoracic limb brachial intumescence at each time point studied, between 15 minutes and 24 hours after loading for a panel of candidate genes. It might be expected that activation of MAPK signaling through ERK and JNK in ulnar bone would be associated with up-regulation of c-fos and c-jun expression in DRG sensory neurons. However, this was not supported by our data. It is also possible the signaling timeline in bone is different than the timeline in neuronal tissue. Recent work by Tomlinson and others has provided more support for the role of sensory nerves in functional adaptation of the skeleton. NGF expression was increased in osteocalcin-expressing cells from the ulnar bone surface of mice at 1 and 3 hours after mechanical loading [39]. Similarly, mRNA expression of NGF from the central portion of the ulna by qRT-PCR was increased at 3 and 24 hours in loaded limbs [39]. We were unable to identify similar correlations of NGF gene expression with bone signaling in our study. Further work studying gene expression in the ulna diaphysis in our rat model appears warranted.

In a pilot study, the yield of protein for Western blot analysis from the cervical intumescence DRG in rats was limited. Consequently, we chose to pursue analysis of gene expression from neuronal tissue using qRT-PCR to examine downstream products from relevant signaling pathways. Use of more sophisticated and comprehensive methods for detecting RNA products such as microarrays or RNA-Seq may be useful to analyze the transcriptome in DRG, periosteum, and bone from loaded and contralateral limbs. Upregulation of Wnt signaling among other genes at 3 hours and at 24 hours in cortical bone has been demonstrated using this approach [58]. Ultimately, protein expression would need to be assessed to confirm changes in gene expression affect the target protein. Since the periosteum is densely innervated with sensory nerve fibers, examination of this tissue in the region of the most prominent adaptive change may also provide useful information on the neuronal contribution to bone adaptation [59].

Several limitations of this study exist. The time course experiment was chosen to identify time points with increased signaling activity in various pathways. Activation of signaling can be transient. Consequently, peaks of signaling activity may occur in between or beyond the selected time points used in the study. A trend towards increased activity was noted at several time points. However, sample sizes precluded identification of additional significant findings. Post hoc power analysis revealed a group sizes of >20 animals per time point would be needed to achieve significance for the effect of loading on these trends. Selected pathways were studied based on previous *in vitro* work. Other pathways, such as Sost/Sclerostin, Wnt/ β catenin, or NGF/TrkA signaling [28,39,58], are also important for load-induced bone formation and could be studied in more detail *in vivo* using our experimental approach.

In conclusion, our findings from this study extend knowledge of bone mechanotransduction beyond results of *in vitro* models. It is clear the skeletal responses to mechanical loading are complex, particularly when considering the whole animal model in which neurovascular responses to bone loading are intact. Signaling by the MAPK pathway appears to be an important early component to bone mechanotransduction, but the role of other signaling pathways and sensory innervation needs further investigation. Further characterization of the molecular events involved in functional adaptation using this model is needed in future work.

## Supporting information

S1 File Study data is provided in the supporting excel file data file. (XLSX)

## References

[1] Goodman CA, Hornberger TA, Robling AG. Bone and skeletal muscle: Key players in mechanotransduction and potential overlapping mechanisms. Bone. 2015;80: 24–36. doi: 10.1016/j.bone.2015.04.014 26453495 10.1016/j.bone.2015.04.014 PMC4600534

[2] Riddle RC, Donahue HJ. From streaming-potentials to shear stress: 25 years of bone cell mechanotransduction. J Orthop Res. 2009;27: 143–149. doi: 10.1002/jor.20723 18683882 10.1002/jor.20723

[3] Huang H, Kamm RD, Lee RT. Cell mechanics and mechanotransduction: pathways, probes, and physiology. Am J Physiol Cell Physiol. 2004;287: C1–11. doi: 10.1152/ajpcell.00559.2003 15189819 10.1152/ajpcell.00559.2003

[4] Ruff C, Holt B, Trinkaus E. Who's afraid of the big bad Wolff?: ‘Wolff's law’ and bone functional adaptation. Am J Phys Anthropol. 2006;129: 484–498. doi: 10.1002/ajpa.20371 16425178 10.1002/ajpa.20371

[5] McNamara LM, Majeska RJ, Weinbaum S, Friedrich V, Schaffler MB. Attachment of osteocyte cell processes to the bone matrix. Anat Rec. 2009;292: 355–363. 10.1002/ar.20869 PMC2861341 19248169

[6] Palumbo C. A three-dimensional ultrastructural study of osteoid-osteocytes in the tibia of chick embryos. Cell Tissue Res. 1986;246: 125–131. 3779795 10.1007/BF00219008

[7] Tatsumi S, Ishii K, Amizuka N, Li M, Kobayashi T, Kohno K, et al Targeted ablation of osteocytes induces osteoporosis with defective mechanotransduction. Cell Metab. 2007;5: 464–475. doi: 10.1016/j.cmet.2007.05.001 17550781 10.1016/j.cmet.2007.05.001

[8] Schaffler MB, Cheung W-Y, Majeska R, Kennedy O. Osteocytes: master orchestrators of bone. Calcif Tissue Int. 2013;94: 5–24. doi: 10.1007/s00223-013-9790-y 24042263 10.1007/s00223-013-9790-y PMC3947191

[9] Bonewald LF, Johnson ML. Osteocytes, mechanosensing and Wnt signaling. Bone. 2008;42: 606–615. doi: 10.1016/j.bone.2007.12.224 18280232 10.1016/j.bone.2007.12.224 PMC2349095

[10] Frost HM. A determinant of bone architecture. The minimum effective strain. Clin Orthop Relat Res. 1983;175: 286–292. 6839601

[11] Rubin CT, Lanyon LE. Dynamic strain similarity in vertebrates; an alternative to allometric limb bone scaling. J Theor Biol. 1984;107: 321–327. 6717041 10.1016/s0022-5193(84)80031-4

[12] Buckley MJ, Banes AJ, Levin LG, Sumpio BE, Sato M, Jordan R, et al Osteoblasts increase their rate of division and align in response to cyclic, mechanical tension in vitro. J Bone Miner Res. 1988;4: 225–236. 2847838

[13] Nunamaker DM, Butterweck DM, Provost MT. Fatigue fractures in thoroughbred racehorses: Relationships with age, peak bone strain, and training. J Orthop Res. 1990;8: 604–611. doi: 10.1002/jor.1100080417 2355300 10.1002/jor.1100080417

[14] Burr DB, Milgrom C, Fyhrie DP, Forwood MR, Nyska M, Finestone A, et al In vivo measurement of human tibial strains during vigorous activity. Bone. 1996;18: 405–410. 8739897 10.1016/8756-3282(96)00028-2

[15] Cowin SC. On mechanosensation in bone under microgravity. Bone. 1998;22: 119S–125S. 9600767 10.1016/s8756-3282(98)00011-8

[16] Burr DB, Forwood MR, Fyhrie DP. Bone microdamage and skeletal fragility in osteoporotic and stress fractures. J Bone Miner Res. 1997;12: 6–15. doi: 10.1359/jbmr.1997.12.1.6 9240720 10.1359/jbmr.1997.12.1.6

[17] Burr DB. Bone, exercise, and stress fractures. Exerc Sport Sci Rev. 1997;25: 171–194. 9213092

[18] Muir P, Johnson KA, Ruaux-Mason CP. In vivo matrix microdamage in a naturally occurring canine fatigue fracture. Bone. 1999;25: 571–576. 10574577 10.1016/s8756-3282(99)00205-7

[19] Gallea S, Lallemand F, Atfi A, Rawadi G, Ramez V, Spinella-Jaegle S, et al Activation of mitogen-activated protein kinase cascades is involved in regulation of bone morphogenetic protein-2-induced osteoblast differentiation in pluripotent C2C12 cells. Bone. 2001;28: 491–498. 11344048 10.1016/s8756-3282(01)00415-x

[20] Jessop HL, Rawlinson SC, Pitsillides AA, Lanyon LE. Mechanical strain and fluid movement both activate extracellular regulated kinase (ERK) in osteoblast-like cells but via different signaling pathways. Bone. 2002;31: 186–194. 12110433 10.1016/s8756-3282(02)00797-4

[21] Kyono A, Avishai N, Ouyang Z, Landreth GE, Murakami S. FGF and ERK signaling coordinately regulate mineralization-related genes and play essential roles in osteocyte differentiation. J Bone Miner Metab. 2011;30: 19–30. doi: 10.1007/s00774-011-0288-2 21678127 10.1007/s00774-011-0288-2 PMC3192226

[22] Sunters A, Armstrong VJ, Zaman G, Kypta RM, Kawano Y, Lanyon LE, et al Mechano-transduction in osteoblastic cells involves strain-regulated estrogen receptor alpha-mediated control of insulin-like growth factor (IGF) I receptor sensitivity to ambient IGF, leading to phosphatidylinositol 3-kinase/AKT-dependent Wnt/LRP5 receptor-independent activation of beta-catenin signaling. J Biol Chem. 2010;285: 8743–8758. doi: 10.1074/jbc.M109.027086 20042609 10.1074/jbc.M109.027086 PMC2838297

[23] Niziolek PJ, Murthy S, Ellis SN, Sukhija KB, Hornberger TA, Turner CH, et al Rapamycin impairs trabecular bone acquisition from high-dose but not low-dose intermittent parathyroid hormone treatment. J Cell Physiol. 2009;221: 579–585. doi: 10.1002/jcp.21887 19639601 10.1002/jcp.21887 PMC2755650

[24] Millie H-F. Signal transduction and mechanical stress. Sci STKE. 2004;2004: RE12 doi: 10.1126/stke.2492004re12 15353762 10.1126/stke.2492004re12

[25] Liedert A, Kaspar D, Blakytny R, Claes L, Ignatius A. Signal transduction pathways involved in mechanotransduction in bone cells. Biochem Biophys Res Commun. 2006;349: 1–5. doi: 10.1016/j.bbrc.2006.07.214 16930556 10.1016/j.bbrc.2006.07.214

[26] Franceschi RT, Xiao G, Jiang D, Gopalakrishnan R, Yang S, Reith E. Multiple signaling pathways converge on the Cbfa1/Runx2 transcription factor to regulate osteoblast differentiation. Connect Tissue Res. 2013;44: 109–116. PMC3564252 12952183

[27] Robling AG, Turner CH. Mechanical signaling for bone modeling and remodeling. Crit Rev Eukaryot Gene Expr. 2009;19: 319–338. 19817708 10.1615/critreveukargeneexpr.v19.i4.50 PMC3743123

[28] Robling AG, Niziolek PJ, Baldridge LA, Condon KW, Allen MR, Alam I, et al Mechanical stimulation of bone in vivo reduces osteocyte expression of Sost/sclerostin. J Biol Chem. 2008;283: 5866–5875. doi: 10.1074/jbc.M705092200 18089564 10.1074/jbc.M705092200

[29] Schriefer JL, Warden SJ, Saxon LK, Robling AG, Turner CH. Cellular accommodation and the response of bone to mechanical loading. 2005;38: 1838–1845. 10.1016/j.jbiomech.2004.08.017 16023471

[30] Duncan RL, Turner CH. Mechanotransduction and the functional response of bone to mechanical strain. Calcif Tissue Int. 1995 ed. 1995;57: 344–358. 8564797 10.1007/BF00302070

[31] Gross TS, Edwards JL, McLeod KJ, Rubin CT. Strain gradients correlate with sites of periosteal bone formation. J Bone Miner Res. 1997;12: 982–988. doi: 10.1359/jbmr.1997.12.6.982 9169359 10.1359/jbmr.1997.12.6.982

[32] Warden SJ, Hurst JA, Sanders MS, Turner CH, Burr DB, Li J. Bone adaptation to a mechanical loading program significantly increases skeletal fatigue resistance. J Bone Miner Res. 2005;20: 809–816. doi: 10.1359/JBMR.041222 15824854 10.1359/JBMR.041222

[33] Wu Q, Sample SJ, Baker TA, Thomas CF, Behan M, Muir P. Mechanical loading of a long bone induces plasticity in sensory input to the central nervous system. Neurosci Lett. 2009;463: 254–257. doi: 10.1016/j.neulet.2009.07.078 19647783 10.1016/j.neulet.2009.07.078 PMC3424266

[34] Sample SJ, Behan M, Smith L, Oldenhoff WE, Markel MD, Kalscheur VL, et al Functional adaptation to loading of a single bone is neuronally regulated and involves multiple bones. J Bone Miner Res. 2008;23: 1372–1381. doi: 10.1359/jbmr.080407 18410233 10.1359/jbmr.080407 PMC2586809

[35] De Souza RL, Matsuura M, Eckstein F, Rawlinson SC, Lanyon LE, Pitsillides AA. Non-invasive axial loading of mouse tibiae increases cortical bone formation and modifies trabecular organization: a new model to study cortical and cancellous compartments in a single loaded element. Bone. 2005;37: 810–818. doi: 10.1016/j.bone.2005.07.022 16198164 10.1016/j.bone.2005.07.022

[36] Marenzana M, De Souza RL, Chenu C. Blockade of beta-adrenergic signaling does not influence the bone mechano-adaptive response in mice. Bone. 2007;41: 206–215. doi: 10.1016/j.bone.2007.04.184 17543595 10.1016/j.bone.2007.04.184

[37] Sample SJ, Heaton CM, Behan M, Bleedorn JA, Racette MA, Hao Z, et al Role of calcitonin gene-related peptide in functional adaptation of the skeleton. PLoS ONE. 2014;9: e113959 doi: 10.1371/journal.pone.0113959 25536054 10.1371/journal.pone.0113959 PMC4275203

[38] Spencer GJ, Hitchcock IS, Genever PG. Emerging neuroskeletal signalling pathways: a review. FEBS Letters. 2004;559: 6–12. doi: 10.1016/S0014-5793(04)00053-5 14960299 10.1016/S0014-5793(04)00053-5

[39] Tomlinson RE, Li Z, Li Z, Minichiello L, Riddle RC, Venkatesan A, et al NGF-TrkA signaling in sensory nerves is required for skeletal adaptation to mechanical loads in mice. Proc Natl Acad Sci U S A. 2017;114: E3632–E3641. doi: 10.1073/pnas.1701054114 28416686 10.1073/pnas.1701054114 PMC5422802

[40] Boulton TG, Nye SH, Robbins DJ, Ip NY, Radzlejewska E, Morgenbesser SD, et al ERKs: A family of protein-serine/threonine kinases that are activated and tyrosine phosphorylatedin response to insulin and NGF. Cell. 1991;65: 663–675. 2032290 10.1016/0092-8674(91)90098-j

[41] Torrance AG, Mosley JR, Suswillo RF, Lanyon LE. Noninvasive loading of the rat ulna in vivo induces a strain-related modeling response uncomplicated by trauma or periostal pressure. Calcif Tissue Int. 1994;54: 241–247. 8055374 10.1007/BF00301686

[42] Sample SJ, Collins RJ, Wilson AP, Racette MA, Behan MA, Markel MD, et al Systemic effects of ulna loading in male rats during functional adaptation. J Bone Miner Res 2010;25: 2016–2028. doi: 10.1002/jbmr.101 20499374 10.1002/jbmr.101 PMC3153405

[43] O’Neil TK, Duffy LR, Frey JW, Hornberger TA. The role of phosphoinositide 3-kinase and phosphatidic acid in the regulation of mammalian target of rapamycin following eccentric contractions. J Physiol. 2009;587: 3691–3701. doi: 10.1113/jphysiol.2009.173609 19470781 10.1113/jphysiol.2009.173609 PMC2742291

[44] Livak KJ, Schmittgen TD. Analysis of relative gene expression data using real-time quantitative PCR and the 2−ΔΔCT method. Methods. 2001;25: 402–408. doi: 10.1006/meth.2001.1262 11846609 10.1006/meth.2001.1262

[45] Rapanelli M, Lew SE, Frick LR, Zanutto BS. Plasticity in the rat prefrontal cortex: linking gene expression and an operant learning with a computational theory. PLoS ONE. 2010;5: e8656–10. doi: 10.1371/journal.pone.0008656 20111591 10.1371/journal.pone.0008656 PMC2810321

[46] Squillacioti C, De Luca A, Paino S, Langella E, Mirabella N. Effects of castration on the expression of the NGF and TrkA in the vas deferens and accessory male genital glands of the rat. Eur J Histochem. 2009;53: 239–248. 22073361 10.4081/ejh.2009.e29 PMC3167333

[47] Papachristou DJ, Papachroni KK, Basdra EK, Papavassiliou AG. Signaling networks and transcription factors regulating mechanotransduction in bone. Bioessays. 2009;31: 794–804. doi: 10.1002/bies.200800223 19444851 10.1002/bies.200800223

[48] Papachristou D, Pirttiniemi P, Kantomaa T, Agnantis N, Basdra EK. Fos- and Jun-related transcription factors are involved in the signal transduction pathway of mechanical loading in condylar chondrocytes. Eur J Orthod. 2006;28: 20–26. doi: 10.1093/ejo/cji101 16373449 10.1093/ejo/cji101

[49] Saxon LK, Robling AG, Alam I, Turner CH. Mechanosensitivity of the rat skeleton decreases after a long period of loading, but is improved with time off. Bone. 2005;36: 454–464. doi: 10.1016/j.bone.2004.12.001 15777679 10.1016/j.bone.2004.12.001

[50] McKenzie JA, Silva MJ. Comparing histological, vascular and molecular responses associated with woven and lamellar bone formation induced by mechanical loading in the rat ulna. Bone. 2011;48: 250–258. doi: 10.1016/j.bone.2010.09.005 20849995 10.1016/j.bone.2010.09.005 PMC3021598

[51] Greenblatt MB, Shim J-H, Glimcher LH. Mitogen-activated protein kinase pathways in osteoblasts. Annu Rev Cell Dev Biol. 2013;29: 63–79. doi: 10.1146/annurev-cellbio-101512-122347 23725048 10.1146/annurev-cellbio-101512-122347

[52] Plotkin LI, Mathov I, Aguirre JI, Parfitt AM, Manolagas SC, Bellido T. Mechanical stimulation prevents osteocyte apoptosis: requirement of integrins, Src kinases, and ERKs. Am J Physiol Cell Physiol. 2005;289: C633–43. doi: 10.1152/ajpcell.00278.2004 15872009 10.1152/ajpcell.00278.2004

[53] Robling AG, Castillo AB, Turner CH. Biomechanical and molecular regulation of bone remodeling. Annu Rev Biomed Eng. 2006;8: 455–498. doi: 10.1146/annurev.bioeng.8.061505.095721 16834564 10.1146/annurev.bioeng.8.061505.095721

[54] Wadhwa S, Godwin SL, Peterson DR, Epstein MA, Raisz LG, Pilbeam CC. Fluid flow induction of cyclo-oxygenase 2 gene expression in osteoblasts is dependent on an extracellular signal–regulated kinase signaling pathway. J Bone Miner Res. 2002;17: 266–274. doi: 10.1359/jbmr.2002.17.2.266 11811557 10.1359/jbmr.2002.17.2.266

[55] Boyle WJ, Simonet WS, Lacey DL. Osteoclast differentiation and activation. Nature. 2003;423: 337–342. doi: 10.1038/nature01658 12748652 10.1038/nature01658

[56] Mosley JR, March BM, Lynch JA, Lanyon LE. Strain magnitude related changes in whole bone architecture in growing rats. Bone. 1997;20: 191–198. 9071468 10.1016/s8756-3282(96)00385-7

[57] Kotha SP, Hsieh YF, Strigel RM, Müller R, Silva MJ. Experimental and finite element analysis of the rat ulnar loading model–correlations between strain and bone formation following fatigue loading. J Biomech 2004;37:541–548. doi: 10.1016/j.jbiomech.2003.08.009 14996566 10.1016/j.jbiomech.2003.08.009

[58] Kelly NH, Schiumenti JC, Ross FP, van der Meulen MCH. Transcription profiling of cortical versus cancellous bone from mechanically-loaded murine tibia reveals differential gene expression. Bone 2016;86:22–29. doi: 10.1016/j.bone.2016.02.007 26876048 10.1016/j.bone.2016.02.007 PMC4833881

[59] Hill EL, Elde R. Distribution of CGRP-, VIP-, D beta H-, SP-, and NPY-immunoreactive nerves in the periosteum of the rat. Cell Tissue Res. 1991;264: 469–480. 1714353 10.1007/BF00319037

